# Assessing Engagement of Adolescents and Young Adults (AYA) in HIV Research: A Multi-method Analysis of a Crowdsourcing Open Call and Typology of AYA Engagement in Sub-Saharan Africa

**DOI:** 10.1007/s10461-022-03786-3

**Published:** 2022-07-12

**Authors:** Kadija M. Tahlil, Laura Rachal, Titi Gbajabiamila, Ucheoma Nwaozuru, Chisom Obiezu-Umeh, Takhona Hlatshwako, Mandikudza Tembo, Nicola Willis, Carine Oum Nyagog, Susan Vorkoper, Rachel Sturke, Nora E. Rosenberg, Victor Ojo, Isaac Moses, Nadia Ahmed, Kristin Beima-Sofie, Sarah T. Roberts, Brenda Kateera, Eleanor Namisoke-Magongo, Michael T. Mbizvo, Juliet Iwelunmor, Oliver Ezechi, Joseph D. Tucker

**Affiliations:** 1 Department of Epidemiology, Gillings School of Global Public Health, University of North Carolina at Chapel Hill, Chapel Hill, NC USA; 2 School of Medicine, University of North Carolina at Chapel Hill, Chapel Hill, NC USA; 3 Clinical Sciences Department, Nigerian Institute of Medical Research, Lagos, Nigeria; 4 Department of Behavioral Science and Health Education, Saint Louis University, Saint Louis, MO USA; 5 Department of Health Policy and Management, Gillings School of Global Public Health, University of North Carolina at Chapel Hill, Chapel Hill, NC USA; 6 MRC Tropical Epidemiology Group, London School of Hygiene and Tropical Medicine, London, UK; 7 Africaid, Harare, Zimbabwe; 8 Gender Data Journalists Network, Yaoundé, Cameroon; 9 Fogarty International Center, National Institutes of Health, Bethesda, MD USA; 10 Department of Health Behavior, Gillings School of Global Public Health, University of North Carolina at Chapel Hill, Chapel Hill, NC USA; 11 Mortimer Market Centre, Central North West London NHS Trust, London, UK; 12 Department of Global Health, University of Washington, Seattle, WA USA; 13 Women’s Global Health Imperative, RTI International, Berkeley, CA USA; 14 AIDS Healthcare Foundation (AHF) Rwanda, Kigali, Rwanda; 15 Pediatric and Adolescent HIV Care and Treatment, AIDS Control Program, Ministry of Health, Kampala, Uganda; 16 Population Council, Lusaka, Zambia; 17 Institute of Global Health and Infectious Diseases, University of North Carolina at Chapel Hill, Chapel Hill, NC USA; 18 Faculty of Infectious and Tropical Diseases, London School of Hygiene and Tropical Medicine, London, UK; 19 University of North Carolina School of Medicine, University of North Carolina at Chapel Hill, Chapel Hill, NC 27599 USA

**Keywords:** Adolescents and young people, Crowdsourcing, Africa, Engagement, Typology

## Abstract

Engagement of adolescents and young adults (AYA) in HIV research is increasing in many settings. We organized a crowdsourcing open call to solicit examples of how AYA have been engaged in HIV research in Africa and to develop an engagement typology. We formed a steering committee, promoted the open call, organized judging and recognized finalists. We used a multi-methods approach to identify emerging themes and measure engagement. We received 95 entries from individuals in 15 countries; 74 met the eligibility criteria. More than three-quarters of entries were from AYA (55/74, 74%). Four themes characterized AYA engagement: (1) AYA were co-creators in the HIV research process. (2) AYA were involved in community-level capacity building. (3) AYA were co-leaders in minor risk research. (4) AYA used digital methods to enhance engagement. Our open call identified diverse methods of AYA engagement, which can enhance strategies used to reach AYA in African HIV studies.

**Supplementary Information:**

The online version contains supplementary material available at 10.1007/s10461-022-03786-3.

## Introduction

Engagement of adolescents and young adults (AYA) in AYA-focused HIV research is increasingly recognized as essential [1, 2]. While AYA engagement varies, the cornerstone of AYA engagement is working collaboratively with AYA who share common goals and interests through building authentic partnerships, which include mutual respect, inclusive participation, and equitable relationships [3–7]. Both the Joint United Nations Programme on HIV/AIDS (UNAIDS) and the World Health Organization (WHO) have emphasized the importance of AYA engagement in the planning and implementation of health interventions [8, 9]. AYA engagement can empower young people to provide solutions to health problems and be involved in decision-making processes [10]. It can also enhance participation, recruitment, long-term sustainability, intervention relevance and acceptability because AYA researchers are closest to the issues and most informed about the types of health services offered to AYA [2, 5, 7, 11, 12]. For example, the 4 Youth By Youth (4YBY) research project engaged AYA with modest formal training to successfully increase uptake of HIV testing in Nigeria (Box 1) [13, 14]. This example demonstrates the benefits of AYA engagement in the research process, highlighting how AYA engagement may help optimize reach, uptake and sustainability of HIV interventions [15].

Despite benefits, AYA engagement in HIV implementation science research in sub-Saharan Africa is currently limited [1, 10]. Engagement strategies often rely on in-person methods, despite technological advances in many low- and middle-income countries (LMICs) [16, 17]. In addition, few studies describe or measure the extent of AYA engagement. A more nuanced understanding of AYA engagement in Africa can inform meaningful and equitable participation of AYA in the region. This suggests the need for a typology of AYA engagement in research studies. Few studies describe the details and quality of AYA engagement within HIV research [1]. A typology of AYA engagement is needed to characterize the extent of engagement and inform researchers and practitioners [18, 19].

Responding to this unmet need, we used a crowdsourcing open call to solicit creative examples of how AYA (14–24 years old) have been engaged in HIV research across Africa. A crowdsourcing open call involves a group of individuals attempting to solve a problem followed by sharing solutions with the public [20]. Crowdsourcing open calls among AYA in sub-Saharan Africa have been successful in soliciting concepts, images, and videos to create demand for HIV self-testing [13] and inform sexual health policies [21]. We used a youth participatory action research framework to inform the open call design and analysis [22, 23]. The purpose of this paper is to create a typology for AYA engagement in HIV research and identify overarching themes related to AYA engagement.

Box 1 Through the 4YBY project, Victor Ojo, a Nigerian university student, and his AYA team (Team Pharmanaut) responded to a crowdsourcing open call for creative ideas to promote HIV self-testing among Nigerian AYA. Victor’s team was selected to join a designathon, a 3-day training session to build capacity for AYA implementation and leadership. With mentorship, the AYA team piloted their ideas in their community, which resulted in a 55% increase in HIV testing uptake and similar increases in STI testing.

## Methods

### Overview

We organized a crowdsourcing open call in order to directly learn from AYA and others who developed AYA engagement strategies for HIV research studies in sub-Saharan Africa. We focused on people aged 14–24 years old because of the high burden of HIV in sub-Saharan Africa within this age group. Between October 2020 and April 2021, we conducted a crowdsourcing open call following guidance from the Special Programme for Research and Training in Tropical Diseases (TDR) Practical Guide on Crowdsourcing in Health and Health Research [24]. The open call involved: (1) forming a steering and organizing committee; (2) promoting the open call to solicit ideas; (3) evaluating entries across five criteria and recognizing finalists, (4) analyzing data using a multi-methods approach informed by a youth participatory action research framework; and (5) disseminating open call findings.

### Forming a Steering Committee and Organizing Committee

We organized a steering committee of 17 members consisting of infectious disease epidemiologists, clinicians, public health researchers, policy analysts, and organization leaders. Four members (23.5%) of the steering committee were AYA with expertise in medicine, public health, organizational leadership and youth ambassadorship. The committee consisted of members from seven countries—Zambia, Tanzania, Rwanda, Uganda, Nigeria, United Kingdom and United States. The steering committee met bi-monthly to draft the open call for entries, determine strategies to promote the open call, establish the criteria to evaluate entries, decide how to recognize finalists, and create dissemination plans. The organizing committee had 10 public health and medical professionals who oversaw the daily activities of the open call, such as developing the open call website, creating promotional materials, revising the open call for entries, and answering inquiries from potential applicants.

### Promoting the Open Call to Solicit Ideas

**Fig. 1:**
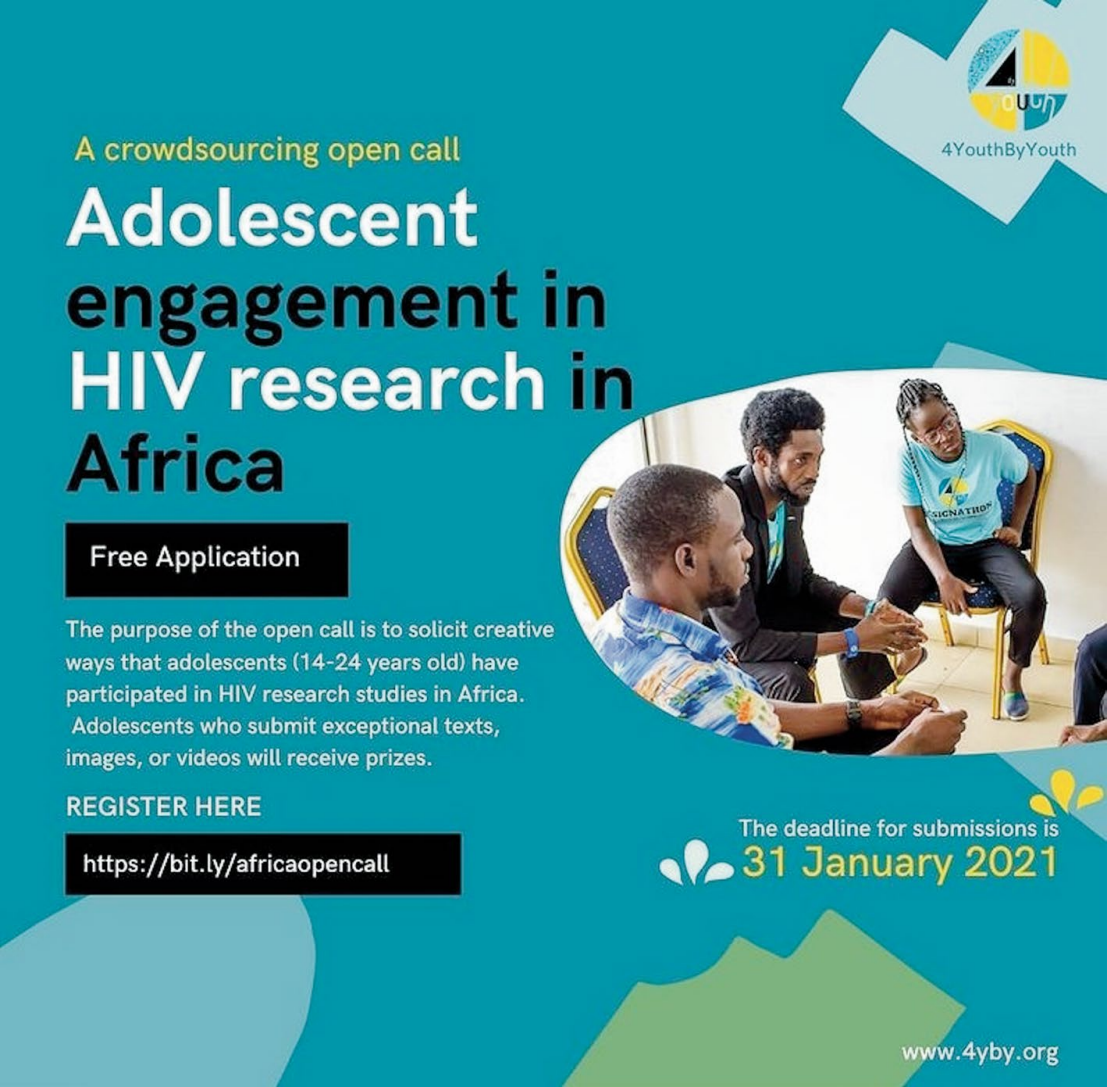
Social media card to promote the crowdsourcing open call

During a 6-week period, we shared the open call with adolescent HIV networks throughout Africa. These networks include the Adolescent HIV Prevention and Treatment Implementation Science Alliance (AHISA), Prevention and Treatment through a Comprehensive Care Continuum for HIV-affected Adolescents in Resource Constrained Settings (PATC3H), and the 4YBY group, which all focus on adolescent HIV implementation science research in LMICs. We went to school districts to advertise the open call and presented at university club or group meetings. We also created and distributed social media cards in English and French on the 4YBY Twitter and Instagram pages (Fig. 1). The 4YBY group, which consists of health professionals and young people interested in improving Nigerian AYA participation in HIV services, was the main host of the open call. The open call was also supported by the Adolescent HIV Prevention and Treatment Implementation Science Alliance (AHISA), a network of researchers, practitioners, and policy leaders focused on AYA HIV implementation science research in Africa [25].

We provided individuals and teams with multiple methods to submit their ideas as texts (maximum 500 words), images (less than 5 MB), or videos (less than 3 min). We did not provide individuals with specific examples of AYA engagement as to not influence their entries, but we clarified the types of ideas we wanted to crowdsource. We accepted entries through email, WhatsApp or Google Forms. Entries were eligible if they were in English or French; if an entry was submitted in a different language, we requested a translated version.

### Evaluating Entries and Recognizing Finalists

The judging process for the open call consisted of three phases. In phase one, two members of the steering committee pre-screened entries for eligibility. Entries were eligible if they addressed the open call prompt and if the country of research was in sub-Saharan Africa. In phase two, a 13-member judging panel, consisting of individuals from the steering committee and 4YBY group, evaluated eligible entries. Judges provided a single overall score for each entry on a scale of 1–10 (with 1 being of the lowest quality and 10 being of the highest quality). This single overall score was determined by the assessing entries in five categories: innovation, feasibility in Africa, clarity, extent of adolescent engagement, and scalability in diverse African settings. Innovation was defined as describing new opportunities for AYA engagement in HIV research. Feasibility was defined as AYA engagement approaches that can be easily implemented. Clarity was defined as entries that were clearly presented in writing or visually through image or video. Extent of AYA engagement was defined as AYA being consulted or involved in decision-making at key stages of research. Scalability was defined as AYA engagement that has the potential to expand throughout Africa. Scores for each entry were tallied, averaged, and then ranked. In phase three, the steering committee met to discuss scored entries, select finalists, and determine the open call prize structure.

The steering committee awarded the top 12 entries with the highest overall mean scores with monetary prizes. The committee identified entries ranked first through fourth as finalists and gave each 500 USD and identified entries ranked fifth through twelfth as semifinalists and gave each 125 USD. The committee awarded every entry with a certificate of commendation in recognition of submission to the open call. We also awarded entries with an overall mean score of 7 points or higher with a certificate of special commendation.

### Analyzing Data from the Open Call

The crowdsourcing open call was guided by the youth participatory action research framework, which encourages young people to learn about social problems and propose potential solutions to those problems [23, 26–28]. We performed descriptive statistics to describe the demographic data of contest submitters including their age, gender, and region of research. Using a modified Hart’s ladder, we categorized the extent of AYA engagement in each entry as absent, minimal, moderate or substantial [1]. Hart’s ladder is a typology that describes different forms of AYA engagement in research projects [29]. Absent AYA engagement was defined as the lack of participatory approaches during the research process. Minimal AYA engagement was defined as AYA being consulted, tasked with specific duties or informed about the research process without having any decision-making power. Moderate AYA engagement was defined as research activities initiated by non-AYA adults with shared decision making with AYA. Substantial AYA engagement was defined as research activities initiated and directed by AYA. We conducted a descriptive thematic analysis to examine the open call data [30, 31]. The analysis proceeded inductively, beginning with developing a codebook that incorporated emergent themes identified during data collection, coding, and then evaluating the coded data to create categories and select illustrative quotes for each category. We created a typology to describe and measure the ways in which AYA have been engaged in HIV research.

### Disseminating Findings

We hosted a webinar for the open call partnered networks [32]. The purpose of the webinar was to share findings from the open call and have the top four finalists present their entries on adolescent engagement (Supplement 1). We also invited the finalists to participate in co-creating this manuscript as co-authors.

### Ethics Approval

We obtained ethical approval to conduct this study from the institutional review board of the Nigerian Institute of Medical Research (Lagos, Nigeria).

## Results

### Demographic and Study Characteristics

We received 95 entries from individuals in 15 sub-Saharan African countries; 74 met the eligibility criteria. Table 1 illustrates the demographic characteristics of the open call participants. Among all eligible participants, a substantial share were male (55.4%), from Western Africa (47.9%), submitted an entry that described HIV prevention research (71.6%) and were researchers (41.9%). The median age of all participants was 23 years (interquartile range 21–25 years; range 14–47 years). AYA and non-AYA adults (25 years or older) contributed 74% and 26% of the eligible entries, respectively. Among all entries, engagement of AYA was absent in 13 (18%), minimal in 27 (36%), moderate in 13 (18%), and substantial in 21 (28%). Fifty-three (72%) studies focused on HIV prevention and 21 (28%) focused on treatment or care.

**Table 1:** Demographic and study characteristics of open call participants in sub-Saharan Africa; 2021 (n = 74)

	All eligible participants (N = 74)	12 highest scoring participants (N = 12)
	n (%)	n (%)
Age (years)		
Median (interquartile range)	23 (21–25)	25 (23–30)
Sex		
Female	33 (44.6)	8 (66.7)
Male	41 (55.4)	4 (33.3)
Missing	0	0
Region of research		
Central Africa	4 (5.5)	1 (8.3)
Eastern Africa	17 (23.3)	2 (16.7)
Southern Africa	17 (23.3)	5 (41.7)
Western Africa	35 (47.9)	4 (33.3)
Missing	1	0
HIV research		
Prevention	53 (71.6)	9 (75)
Treatment or care	21 (28.4)	3 (25)
Role in HIV research		
Participant	14 (18.9)	0 (0)
Researcher	31 (41.9)	6 (50)
Project manager	10 (13.5)	2 (16.7)
Assistant or coordinator	7 (9.5)	1 (8.3)
Multiple roles	12 (16.2)	3 (25)
Extent of engagement		
Absent	13 (17.6)	1 (8.3)
Minimal	27 (36.5)	2 (16.7)
Moderate	13 (17.6)	3 (25)
Substantial	21 (28.3)	6 (50)

### Themes

**Fig. 2:**
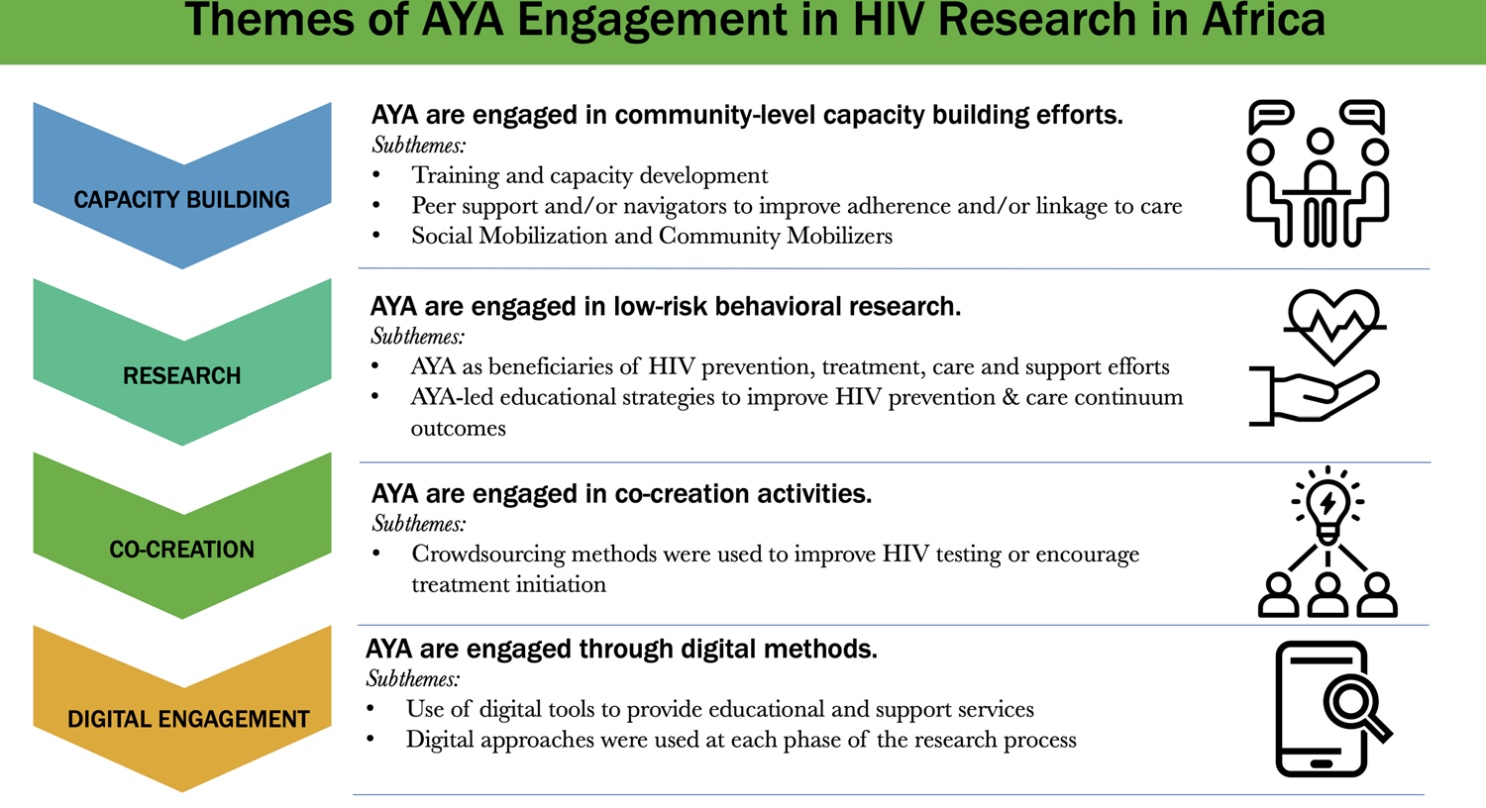
Crowdsourcing open call infographic of AYA engagement in HIV research

Four emergent themes characterized differences among the approaches for AYA engagement: (1) community level capacity building efforts enhanced AYA engagement in HIV research; (2) minor risk behavioral research provided opportunities for AYA leadership; (3) co-creation and AYA-led activities provided mechanisms for substantial AYA engagement; and (4) digital methods utilized by AYA enhanced engagement in research (Fig. 2). All themes had sub-themes that further described the approaches of AYA engagement in HIV research (Supplement 2).

#### Community Level Capacity Building Efforts Enhanced AYA Engagement in HIV Research

We defined community level capacity building efforts as AYA-led approaches to help build skills and knowledge of other AYA and to build community resources and support. Several entries described AYA leading capacity building efforts to support AYA engagement. These entries described how AYA (both AYA living with HIV and those without HIV) were mentored and became advocates for AYA (Supplement 2, Entry #22). The trainings and mentorship provided to AYA equipped them with knowledge and skills required to support AYA in preventing HIV acquisition or transmission (Supplement 2, Entry #49) as well as gender-based violence (Supplement 2, Entry #23).

AYA were also engaged in various forms of training. Some AYA were trained to use digital art to describe and share their understanding of sexual and reproductive health in their communities (Supplement 2, Entry #19) and others were engaged as peer navigators to improve HIV testing and linkage to care (Supplement 2, Entry #68). One entry described AYA training that allowed AYA researchers to advocate for greater access to HIV services (Supplement 2, Entry #47).

#### Minor Risk Behavioral Research Provided Opportunities for AYA Leadership

Enabling AYA to participate in research (e.g. data collection procedures such as focus group discussions and surveys) focused on AYA is a strategy that can reach large numbers of other AYA who can benefit from HIV prevention and treatment services (Supplement 2, Entry #56). In a few entries, AYA led survey research where they engaged other AYA through open contests (Supplement 2, Entry #31). One entry described an open contest to solicit input from large groups of adolescent girls and young women (AGYW) with the purpose of compiling a report about AGYW living with HIV in South Africa (Supplement 2, Entry #14).

Entries differed in the extent to which AYA were allowed to lead research studies. Only a few entries described AYA leading the entire process of the study. These AYA had careers in research and were previously exposed to workshops and trainings that empowered them (Supplement 2, Entry #64). In other entries, AYA has less research experience, but they participated in the planning and evaluation of the study (Supplement 2, Entry #30). Several entries described AYA at the forefront of HIV service promotion to other AYA in their communities and showed AYA as leaders and facilitators in conducting educational activities to improve HIV prevention and care continuum outcomes (Supplement 2, Entry #57).

#### Co-creation and AYA-Led Activities Provided Mechanisms for Substantial AYA Engagement

We define co-creation as participatory approaches that involve AYA as partners and where agendas are created and/or implemented in partnership. In some entries, a co-creation space was created with AYA through open calls. These open calls engaged AYA by soliciting their input on how to improve AYA engagement in HIV testing and treatment programs (Supplement 2, Entries #71, #31). Workshops were also co-creation spaces in which AYA led creative activities and the dissemination of findings (Supplement 2, Entries *#*20, #54).

A few entries described the utilization of crowdsourcing methods to reach a large and diverse group of AYA. Crowdsourcing methods were used to improve HIV testing or encourage treatment initiation. Two specific crowdsourcing methods highlighted in entries were open calls and designathons, which involved AYA in co-creating agendas and implementing research (Supplement 2, Entries #60, #71, #31).

#### Digital Methods Utilized by AYA Enhanced Engagement in Research

Textual data suggested that AYA used digital methods to nurture AYA engagement throughout the lifespan of the study (Table 2). Prominent methods described in the entries include the use of instant messaging applications, especially WhatsApp, among the researchers and AYA. WhatsApp groups were created for AYA to receive health information (e.g. about HIV stigma and discrimination) and psychosocial support (Supplement 2, Entries #03, #49). Facebook and Instagram were also used in sending health information to AYA; one entry described sharing meeting agendas with AYA to prepare them for sessions (Supplement 2, Entry #23). In one study, Facebook and WhatsApp were used as HIV educational tools to communicate with AYA during the COVID-19 pandemic and to help them better comply with prevention measures. (Supplement 2, Entry #36). In other entries, WhatsApp was a platform that allowed AYA to ask questions, submit ideas and receive feedback during open calls (Supplement 2, Entry #71).

**Table 2:** Digital methods to enhance AYA engagement at each stage of the HIV research process

	Stages of research	Digital methods	AYA engagement
1.	Study design	• Electronic articles such as magazine, journal, etc • Using social media for preliminary assessments	• AYA created online content that informed HIV research studies. AYA-authored social media posts can provide a real-time snapshot of youth priorities • AYA engagement with posts can be used to tailor strategies
2.	Recruitment	• Use of websites, messaging applications, social media platforms for recruitment and training	• AYA research assistants recruited research participants through websites, messaging apps, and social media
3.	Implementation	• Group use of social media platforms for: focus group discussions, counseling, education, and survey completion • For data recording and management, to serve as intervention reminder, and data collection-photo/essay/video self-expressions	• AYA decided optimal forms of soliciting information. They also created messages as part of open calls • Data recording methods provided more emotional connection with audiences, reached more AYA (more than text), provided the key information, identified the AYA in participatory video/ photographic images, it provided the views of the AYA directly and reached a larger audience because the data could be recorded on mobile phones
4.	Dissemination	• Social media: websites, and message application distribution • Use of other digital media: video/photo campaigns, audio campaigns	• AYA forwarded messages with study findings through social networks

### Typology

Our typology identified four inter-related levels of AYA engagement (Table 3). These levels are not mutually exclusive and in some settings, higher levels of engagement may not be feasible or desirable. The typology includes an analogy (voice metric to capture the extent of AYA engagement), rationale and power, AYA perspective, and researcher perspective.

**Table 3:** Typology of AYA engagement in research

Extent of engagement	Voice metric	Rationale and power	Examples	AYA perspective	Researcher perspective
Tokenistic	Silent	No power, but with the appearance of genuine input	AYA consultation	Disappointment and frustration, cynicism and lost opportunities	Ethical problems without having any engagement from AYA
Minimal	Muffled	AYA provide input, but overall direction from researchers	Some youth advisory groups	AYA excited about involvement, provides a way to learn about research	Some AYA ideas can be incorporated, maintains researcher control, meets funder requirements for engagement
Moderate	AYA choir or solo	Some coordinated action by AYA, but generally still led by adults	Some qualitative methods (e.g., Focus group discussions), some AYA advisory groups	Builds research experience, introduces AYA to research careers	Provides stronger AYA input, requires some capacity building
Substantial	AYA symphony—complex	Shared power and decision making for AYA; strong voices from youth and an ecosystem to support	AYA-led research, crowdsourcing	Transformative for AYA lives; alters career pathways and life courses; requires more time and integration with school/work if feasible	Potential for disrupting research studies, often requires strong capacity building

#### Tokenistic

Tokenistic engagement occurs when AYA have no voice in any stage of the research. AYA may have been involved as participants or advisors to meet donor requirements, but were not meaningfully involved in any part of the study. In these studies, AYA may be dissatisfied, infuriated, and feel misrepresented. From a researcher perspective, this presents potential ethical problems. The open call descriptions were not sufficiently detailed to understand if engagement was tokenistic.

#### Minimal

AYA participated in some of the research activities and some of their ideas were integrated into the study, but the researchers and the funders directed the research. AYA in this form of engagement are willing and eager to work with the researchers to achieve their objectives. One entry described how adult representatives in the community (e.g. religious leaders, parents, administrators, politicians) were the major stakeholders, while just two representatives for AYA were consulted about condom use among AYA. Information obtained from this entry was disseminated among the AYA through a movie. We determined this level of engagement with AYA as minimal.

#### Moderate

Moderate AYA engagement was observed in entries where AYA set the research agenda, organized training, or designed capacity-building activities but the research process was adult-led. Some education-based entries demonstrated moderate engagement as AYA had some input in the studies and were introduced to careers in research. Other entries described training AYA in research processes and they participated in developing protocols, collecting and analyzing data, and disseminating findings. However, the research remained led and supervised by the adult research teams.

#### Substantial

Substantial engagement exists when AYA and researchers share decision-making power. Entries in this typology were AYA-led and involved social mobilization and community mobilizers. Some entries with substantial AYA engagement involved educational strategies to improve HIV prevention & care continuum outcomes. This form of engagement is evident in entries where AYA co-lead in all the stages of research.

## Discussion

Our crowdsourcing open call solicited many exceptional examples of AYA engagement in HIV research studies across sub-Saharan Africa. We discovered that AYA often lead core elements of study design, implementation, and dissemination. Several minor risk behavioral studies were organized and led by AYA, demonstrating that AYA had the skills and tools for this level of engagement. At the same time, AYA played an important role in building capacity for HIV research, serving as peer mentors and contributing to the training that was necessary for expanded AYA engagement. This study extends the literature by focusing on AYA engagement in Africa, including voices of AYA themselves, and further developing a typology to describe AYA engagement.

Our textual data revealed substantial AYA engagement as part of co-creation activities. One of the more unique methods of AYA involvement amongst our data revealed a common theme of AYA-directed research projects such as crowdsourcing open calls. These participatory projects provided an opportunity for AYA to contribute to promotional materials on gender-based violence, women’s health, and HIV testing. There were also projects that specifically sought to design interventions based on open call ideas through designathons or hackathons. These are sprint-like events where individuals work together for a short period of time to create and present their solutions. Previous crowdsourcing studies in sub-Saharan Africa have solicited AYA to create short scripts that are then developed into films promoting HIV services [33–35] and synthesized AYA ideas as part of designathons [13–15]. Soliciting ideas for promotional awareness as well as using the collective knowledge of AYA to design projects specifically directed at AYA via crowdsourcing methods allow for AYA to be included throughout HIV outreach projects.

Our findings suggest that community-level capacity building enriches AYA engagement in HIV research. For instance, training and mentorship opportunities were provided to select AYA to address practical knowledge gaps, increase motivation and cultivate skills in areas such as communication and leadership. AYA were also involved as peer educators and mentors in programs to facilitate reciprocal learning and dialogue among other AYA in the community. Although existing evidence supports the effectiveness of peer-led interventions for HIV prevention and care among AYA in LIMCs, there are still gaps on how to sustain and scale up these strategies [36–38]. Future research should address critical gaps in the assessment of long-term impact of peer-led capacity building strategies and apply a more comprehensive approach in the evaluation. However, developing capacity building efforts as it relates to AYA and HIV research in Africa provide many benefits including research directed at local scientific and health issues, increased self-efficacy and ownership of local projects, increased interested in research careers and more buy-in from local leadership [39].

We found that many African AYA used diverse digital methods to engage in HIV research studies. This is consistent with other studies in Africa [40–42]. AYA used digital tools to promote communication, education and peer counseling. Examples of this digital engagement included social media messages to promote enrollment, collecting data and consent forms via digital forms, and providing group education and counseling through messaging mobile phone applications. The frequent use of digital methods may have been partly related to COVID-19 measures that prevented or delayed in-person activities. At the same time, many of the AYA engagement strategies predated COVID-19 and will benefit from Africa’s expanding internet coverage. From our open call, instant-messaging platforms were commonly used for participant enrollment and surveys, counseling, education sessions, and sharing results. This is consistent with a broader literature on digital engagement [41, 43, 44].

Our typology of AYA engagement can help to better measure and report engagement in the context of research studies. Researchers can utilize the four approaches (voice metric, rationale and power, AYA perspective and researcher perspective) of the typology to gauge AYA engagement in their studies and reporting on these approaches can inform meaningful participation of AYA. Our open call suggests that while AYA are engaged at various stages of HIV research, expanding AYA engagement in HIV research is still necessary, particularly in the area of designing study interventions. Research training for AYA can be a useful approach to substantially engage AYA in study design, providing the opportunity for AYA to develop research skills and share decision-making power. Among the 74 entries, only one-quarter revealed substantial AYA involvement. The lack of substantial AYA involvement could be attributed to entrants believing AYA were engaged in the interventions and programs described in their entries, but not fully understanding engagement beyond AYA as simply participants in HIV research. Also, higher levels of AYA engagement may not be feasible or desirable in settings with little youth capacity building opportunities or where community members have greater influence on AYA engagement in research. Given that our open call was focused on AYA engagement, the actual frequency of AYA engagement is likely lower. This suggests the need to increase AYA engagement, including creating meaningful opportunities and active participation [3]. Involvement of AYA in AYA-focused research is important. It not only opens up career opportunities and allows for peer education, but it also allows for shared life experiences and peer support to be incorporated into research projects.

Our study has several limitations. First, our open call was not population-based and some AYA engaged in HIV research in sub-Saharan Africa may have not received the open call. At the same time, we partnered with the several networks focused on AYA HIV research, used diverse promotion methods, and allowed submissions using different methods. Second, requiring entries in English or French likely excluded examples of local HIV research engagement and engagement in countries where Portuguese and Spanish are the official languages. Better understanding AYA engagement in local languages may be useful for more contextually appropriate programs. Third, our open call required an individual to have some form of internet access, which likely excluded some low income and/or rural individuals. This likely skewed our engagement descriptions towards individuals focused on digital engagement and teams with better bandwidth. Fourth, our open call may have only solicited examples of AYA engagement that went well. We did not receive entries that described tokenistic engagement and therefore may not have captured the range of AYA engagement across sub-Saharan Africa.

Findings from our crowdsourcing open call have implications for research and policy. From a research perspective, partnering with AYA to identify ways in which AYA are engaged in HIV research studies will result in much needed evidence and insights into improving AYA HIV prevention and care continuum outcomes. HIV clinical trial researchers and program designers are thus encouraged to consider the proposed typology that delineates four distinct approaches to the extent of AYA engagement based on the voice metric, power relationships, AYA perspective and researcher perspective. Reporting on these four metrics could facilitate robust and more inclusive research into the effectiveness of peer-led approaches in research. Furthermore, future measurements of engagement should assess whether AYA are included and contribute to manuscript development for published papers. Our open call findings also have implications for implementation science. Many of the digital forms of AYA engagement we identified could be used to strengthen AYA input on research studies in sub-Saharan Africa. In addition, a crowdsourcing open call could be used to identify implementation science best practices.

## Conclusions

Our crowdsourcing open call identified diverse methods of AYA engagement, providing a strong foundation to create a new typology of engagement. The findings enhance our understanding of the extent and methods of AYA involvement in HIV research and can be used to enhance AYA HIV engagement across the life of research studies in sub-Saharan Africa.

## Supplementary Information

Below is the link to the electronic supplementary material.

Supplementary file1 (DOCX 393 KB)

## Data Availability

Data are available from the corresponding author on request.
