# Supplementary material for: Assessing Engagement of Adolescents and Young Adults (AYA) in HIV Research: A Multi-method Analysis of a Crowdsourcing Open Call and Typology of AYA Engagement in Sub-Saharan Africa
Source: AIDS Behav. 2022 Jul 12;27(Suppl 1):116–27. doi: 10.1007/s10461-022-03786-3 (PMC9277597; doi:10.1007/s10461-022-03786-3)
Supplement: Supplementary file 1 — Supplementary file1 (DOCX 393 KB) [file 10461_2022_3786_MOESM1_ESM.docx]

SUPPLEMENT 1. Engagement of adolescent and young adults (AYA) in HIV research as described by the top four finalists

SUPPLEMENT 2. Illustrative quotes for each theme of AYA engagement

SUPPLEMENT 2. Illustrative quotes for each theme of AYA engagement (continued)
